# Impaled Unexploded Mortar Shell Injury during Iran-Iraq War 1980–1988

**DOI:** 10.34172/aim.2022.67

**Published:** 2022-06-01

**Authors:** Ahmadreza Afshar, Ali Tabrizi

**Affiliations:** ^1^Department of Orthopedics, Imam Khomeini Hospital, Urmia University of Medical Sciences, Urmia, Iran

## Dear Editor,

**Figure 1:**
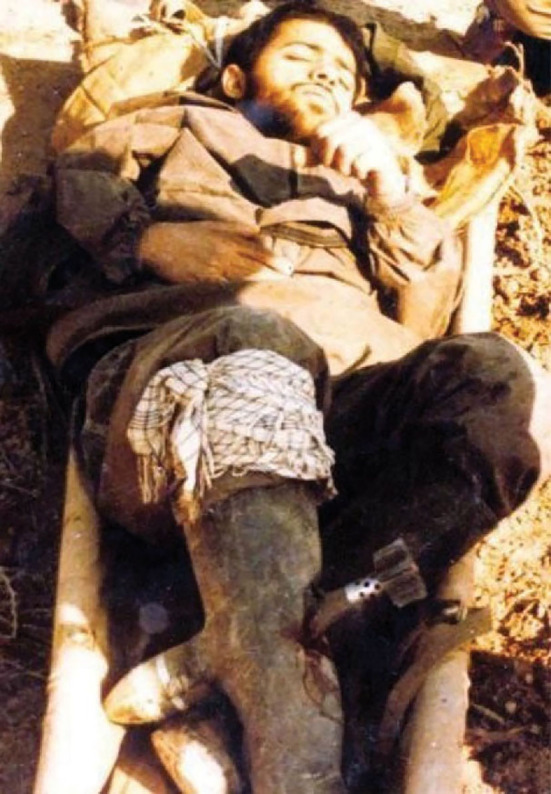
An Impaled Unexploded 60 mm Mortar Shell in the Right Leg of an Iranian Combatant during the Iran-Iraq War 1980–1988.

 On the occasion of the 41th anniversary of the Iran-Iraq war 1980–1988, on September 22, 2021, the Iranian Academy of Medical Sciences honored “Dr. Iraj Mahjoub”, a general surgeon, who in a voluntary and courageous act in the Iran-Iraq war front line removed an impaled unexploded mortar that might have exploded at any moment from a wounded combatant. On January 12, 1986, the right leg of an Iranian combatant was hit with a 60mm mortar shell which did not detonate. Dr. Iraj Mahjoub removed the impaled unexploded mortar shell in an ambulance under general anesthesia. There was no neurovascular injury. Plain radiographs demonstrated a fibular fracture while the tibia was intact (Figure 1).

**Figure 2:**
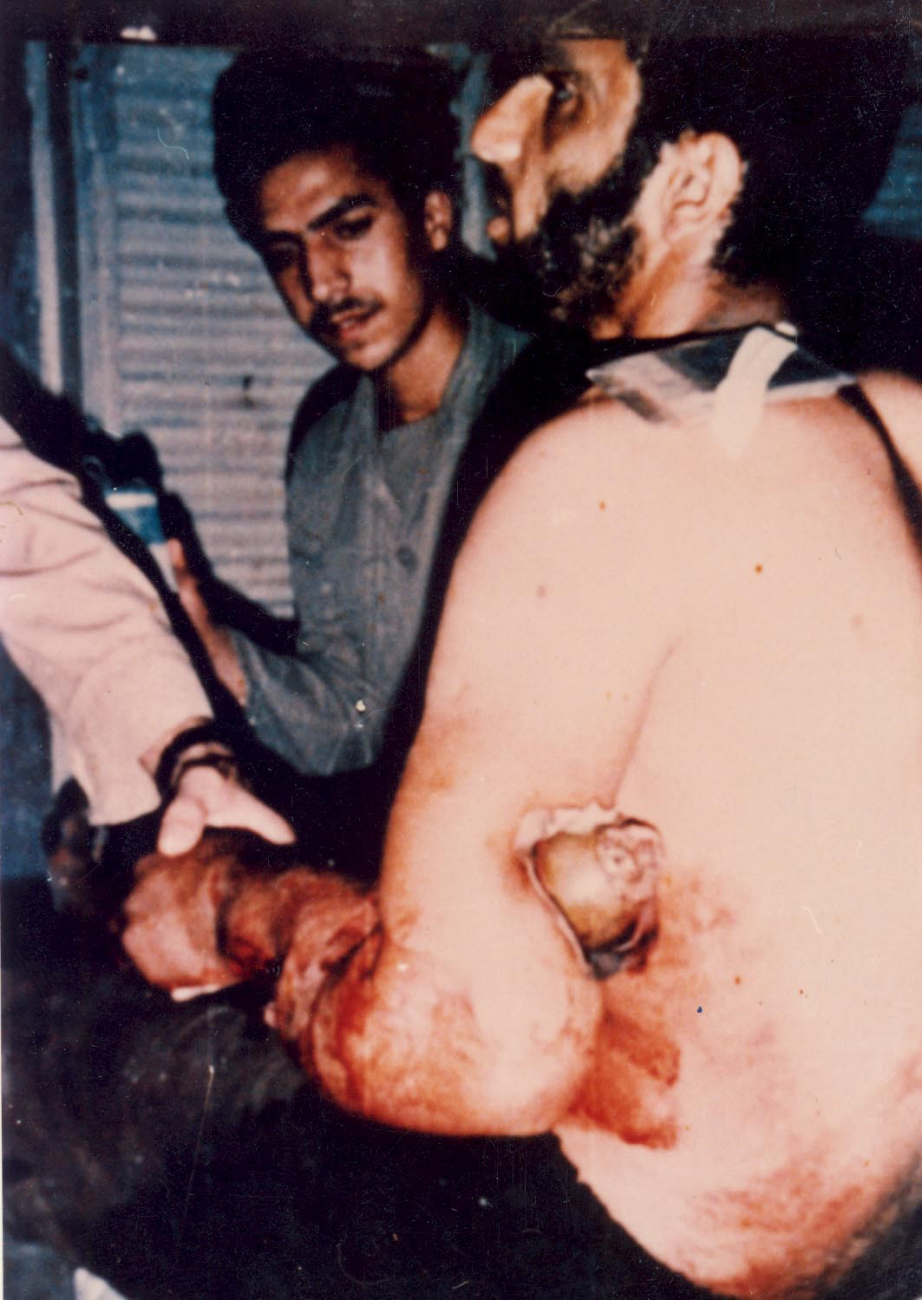
An Impaled Unexploded 60 mm Mortar Shell in the Left Upper Limb of an Iranian Combatant during the Iran-Iraq War 1980–1988.

 The above-mentioned report incited us to present another case of impaled unexploded mortar shell during the Iran-Iraq war. On August 24, 1985, the left upper limb of an Iranian combatant was hit with a 60mm mortar shell while he was talking on a communicating device. The unexploded mortar sewed the left forearm and arm together (Figure 2). Doctor Mohajer removed the impaled unexploded mortar shell in the operating room under general anesthesia. Plain radiographs demonstrated that the humerus, radius and ulnar bones were fractured; however, there was no neurovascular injury. The patient was further treated by Dr. Shojaedin Sheikholeslamzadeh.^1^

 Treatment of war wounds has been a significant topic since ancient times.^2^ There is little to be found in the literature regarding impaled unexploded ordnance (UXO) injuries.^3-5^ In a review of 50 years of military experience and case reports from World War II, Vietnam, and Somalia, Lein et al found 36 patients with impaled UXO. Twelve out of 36 patients had injuries from impaled unexploded 48mm to 82 mm mortar ordnances. (3) In 2005, Oh et al recognized six further known cases of impaled UXOs during the US wars in Afghanistan (four) and Iraq (two).^4^ There was one additional case of impaled UXO in Pakistan.^5^

 Impaled UXO may be a risk to care providers, equipment and surroundings of the victim. Although there is no reported incident of explosion during transportation, preparation, or removal of an impaled UXO, unnecessary individuals must leave the scene. Combustible agents such as oxygen, alcohol-based solutions and combustible volatile anesthetics must be removed from the operating theater.^3,4^

 A review of the reported cases demonstrates that the impaled UXO were some sort of propelled explosive devices such as mortars, rocket propelled grenades (RPG) and rifle-launched grenades. These ordnances basically include a propulsion system, a trigger mechanism, and a main explosive component. Appropriate management requires thorough knowledge of the triggering mechanisms because an inadvertent bypass of the safety mechanism or a malfunction may trigger the ordnance to explode.^3,4^

 All impaled ordnances must be considered armed or activated. There are a variety of triggering mechanisms for explosive ordnances which are usually located at the tip of the main explosives. A mechanical pressure on the percussion cap may trigger a firing mechanism that explodes the device upon impact. Piezoelectric crystals may be another arming mechanism. A piezoelectric crystal at the nose of the projectile generates electricity discharges upon direct contact that detonate the main explosives. Piezoelectric discharges can be also released upon exposure to electricity, light and thermal energy. Therefore, repositioning the patient, direct intense light on the crystal, applying an electric current to the device (for example, careless use of electrocautery) may discharge the triggers to ignite the main explosives. Use of mechanical blood warmers, monitors, blood pressure gauges and infusers or pumps must be reduced in order to minimize the risk of static electrical discharge. Mechanical non-powered manual saws and drills rather than saws and drills that use electricity and pneumatics must be used because of concerns about discharge and vibrations.^3,4^

 This letter emphasizes that there is a risk with impaled UXO injuries. Each reported case illustrates the importance of cataloguing experiences learned during crisis situation and bringing further insight to management of this very unusual and serious type of injury.
